# The impact of temperature and precipitation on blacklegged tick activity and Lyme disease incidence in endemic and emerging regions

**DOI:** 10.1186/s13071-016-1894-6

**Published:** 2016-11-25

**Authors:** James C. Burtis, Patrick Sullivan, Taal Levi, Kelly Oggenfuss, Timothy J. Fahey, Richard S. Ostfeld

**Affiliations:** 1 Department of Natural Resources, Cornell University, Fernow Hall, Ithaca, NY USA; 2 Department of Fisheries and Wildlife, Oregon State University, Corvallis, OR USA; 3 Cary Institute of Ecosystem Studies, 2801 Sharon Turnpike, Millbrook, NY USA

**Keywords:** *Ixodes scapularis*, Lyme disease, Temperature, Precipitation, United States

## Abstract

**Background:**

The incidence of Lyme disease shows high degrees of inter-annual variation in the northeastern United States, but the factors driving this variation are not well understood. Complicating matters, it is also possible that these driving factors may vary in regions with differing histories of Lyme disease endemism. We evaluated the effect of the number of hot (T > 25 °C), dry (precipitation = 0) days during the questing periods of the two immature *Ixodes scapularis* life stages (larval and nymphal) on inter-annual variation in Lyme disease incidence between 2000 and 2011 in long-term endemic *versus* recently endemic areas. We also evaluated the effect of summer weather on tick questing activity and the number of ticks found on small mammals between 1994 and 2012 on six sites in Millbrook, NY.

**Results:**

The number of hot, dry days during the larval period of the previous year did not affect the human incidence of Lyme disease or the density of questing nymphs the following season. However, dry summer weather during the nymphal questing period had a significant negative effect on the incidence of Lyme disease in the long-term endemic areas, and on the density of questing nymphs. Summer weather conditions had a more pronounced effect on actively questing *I. scapularis* collected *via* dragging than on the number of ticks found feeding on small mammals. In recently endemic areas Lyme disease incidence increased significantly over time, but no trend was detected between disease incidence and dry summer weather.

**Conclusions:**

Recently endemic regions showed an increase in Lyme disease incidence over time, while incidence in long-term endemic regions appears to have stabilized. Only within the stabilized areas were we able to detect reduced Lyme disease incidence in years with hot, dry summer weather. These patterns were reflected in our field data, which showed that questing activity of nymphal *I. scapularis* was reduced by hot, dry summer weather.

## Background

Lyme disease is the most common vector-borne disease in the United States [1]. Many studies have attempted to improve our understanding of the factors driving its spread and amplification in new areas [2, 3] as well as the habitat suitability of new areas in the United States and Canada for *Ixodes scapularis* [4–6], the primary vector for the Lyme disease spirochete (*Borrelia burgdorferi*). Correlative models have explored the effect of climatic factors on Lyme disease incidence averaged across expansive geographical regions, most commonly states in the U.S.A. [7, 8]. Unfortunately, there are large variations in incidence within many states [9], and the factors that drive inter-annual variation in Lyme disease incidence vary spatially [10]. Specifically, underlying factors such as physician and public awareness have a strong impact on the reporting of Lyme disease cases in emerging areas [11, 12], and data collected at a coarse scale (State) may not reveal patterns that exist at finer scales (County).

Climate and weather conditions are likely to play a role in the incidence of Lyme disease because the demography and behavior of *I. scapularis* are sensitive to variation in temperature and precipitation [8, 13]. However, few studies have attempted to connect these weather and climate effects to patterns in the human incidence of Lyme disease [10]. There is strong evidence that Lyme disease and tick populations are spreading from two epicenters in the United States, one in the upper Midwest, and another the Northeast [14, 15]. Some have argued that the geographical spread of Lyme disease is caused, at least in part, by climate warming trends [16]. However, other factors might contribute to the spread of Lyme disease, including facilitation of the expansion of *I. scapularis* populations by vertebrate hosts [17], and amplification of *B. burgdorferi* infection through vector and host communities [18, 19]. Possible effects of climate and weather on disease incidence in these newly emerging areas may be difficult to detect due to these confounding factors. In contrast, the effects of weather and climate on Lyme disease incidence in areas that were invaded decades ago might be less subject to confounding variables. Differences in the focal area under study (expanding *vs* long-term endemic regions) may explain some of the variation in results between studies that have explored the effect of weather and climate on inter-annual variation in human cases of Lyme disease [20].

The effect of specific climatic variables on *I. scapularis* density, survival, and behavior has been thoroughly investigated [21–25]. The effect of relative humidity on *I. scapularis* survival is well documented, with significantly reduced survival in low humidity environments [26, 27]. Duration of exposure to dry conditions is an important factor in determining *I. scapularis* mortality, with longer periods of exposure leading to significantly higher rates of mortality [28]. Many species of tick will modify their behavior to avoid desiccation [29]. One of the most commonly observed behaviors is that *I. scapularis* will quest at a lower height when temperatures are high and relative humidity is low [22], probably because of the need to descend to moist microhabitats for rehydration. This lower questing height reduces the probability that ticks will come into contact with large vertebrate hosts [30, 31], including humans. The effect of atmospheric saturation deficit on tick behavior has been studied in *I. ricinus* [32], a tick species that is closely related to *I. scapularis* and is the primary vector for Lyme disease in Europe. Atmospheric humidity has the strongest impact on *I. ricinus* behavior when temperatures are greater than 24 °C [33, 34]. Furthermore, *I. scapularis* questing behavior peaks at 25 °C with lower levels of activity as the temperature increases [35]. In 2014 Berger et al. [36] found that low relative humidity reduces the seasonal activity of *I. scapularis* nymphs. They also found that early season dry periods could lead to reduced late-season tick populations. We expanded on this research by exploring the effect of summer climate on tick density with an additional metric, small mammal body burdens, and by exploring the effect of summer weather on the human incidence of Lyme disease across a broad region.

We analyzed data collected by the Centers for Disease Control and Prevention (CDC) for annual Lyme disease incidence by county, and a long-term field dataset for tick densities collected at the Cary Institute of Ecosystem Studies [37]. We explored the effect of the number of hot (T > 25 °C) dry (precipitation = 0) days on the human incidence of Lyme disease and *I. scapularis* activity. We targeted our analyses for the activity peaks of two *I. scapularis* life stages: (i) May through July for nymphs (second-instar), and (ii) August through September for larvae (first-instar). The nymphal life stage is responsible for the majority of human infections [38], and recruitment and survival of the larval stage is likely to have a strong effect on the number of nymphs emerging the following year [37, 39]. Additionally, we explored whether the effect of summer weather on inter-annual variation in Lyme disease incidence was consistent between areas with a long history of endemic Lyme disease, and those that have become endemic more recently. We hypothesized that weather effects would be more evident in long-term endemic areas, defined as those areas that have the longest history of reported Lyme cases [9, 40, 41].

We also used field measurements to distinguish whether the effect of summer weather conditions on *I. scapularis* is behavioral, or demographic, affecting long-term survival rates. Behavioral effects could manifest as either lower overall questing activity, lower questing height, or a combination of the two. We used two methods to distinguish between weather effects on questing activity *versus* questing height. If hot, dry weather reduces questing height, we expected to find reduced numbers of questing *I. scapularis* nymphs captured with the tick dragging method as this method is most efficient when nymphal questing height is high [42]. In contrast, if hot, dry weather reduces overall questing activity, we would expect that the number of ticks feeding on small mammals, which are sampling ticks at ground level, would be reduced [31]. If the number of hot, dry days during the previous year’s larval questing period reduces nymphal tick densities or Lyme disease incidence the following summer, then we would conclude that the long-term demographic effects of summer weather are important. Both behavioral and demographic effects can impact human health, but the implications for the long-term effect of climate change on tick-borne diseases may differ.

## Methods

### Lyme disease data

**Fig. 1:**
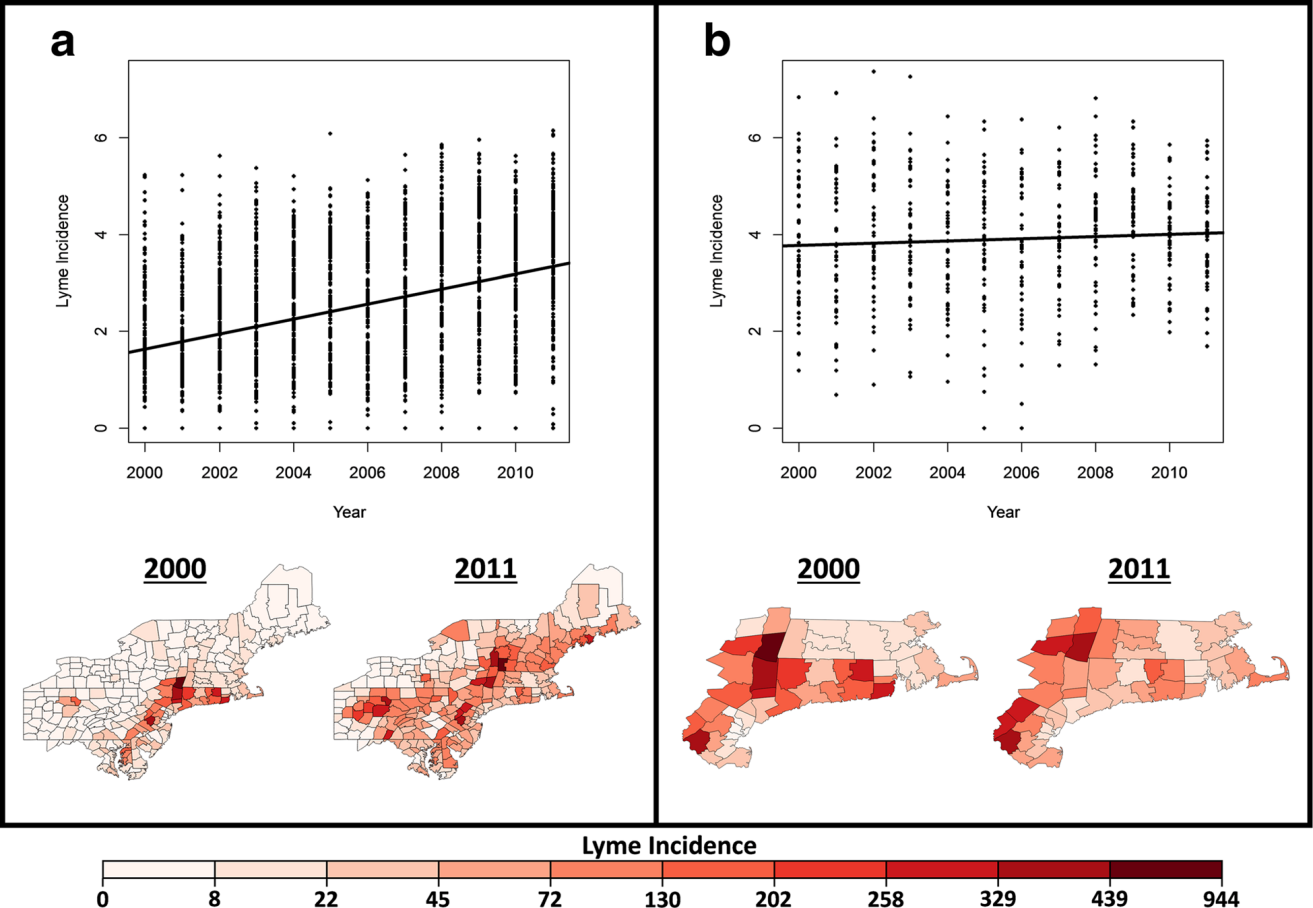
**a** The incidence of Lyme disease in the recently endemic region over time. The scatterplot shows the log of the incidence as time progresses from 2000 to 2011 in the recently endemic region, while the maps show the incidence at the starting point (2000), and ending point (2011) for our entire dataset. **b** The incidence of Lyme disease in the counties of our subset of long-term endemic counties in the Hudson Valley of New York, southern New England and northern New Jersey. The legend represents the incidence of Lyme disease per 100,000 people and the color scheme applies to all four maps

The annual numbers of Lyme disease cases were reported by county between 2000 and 2011 by the CDC [43]. Lyme disease cases are generally under-reported by the CDC [44]. In an effort to address under-reporting the CDC altered their reporting standards in 2008, broadening the criteria for reportable cases. To account for possible under-reporting bias we have included the CDC’s reporting type as a factor in our analyses. Annual Lyme disease case counts are available for all United States counties, but cases are reported in the patient’s county of residence, so in order to reduce reporting errors due to patient travel we focused on the northeastern United States where local incidences are high and > 80% of cases are reported. We split these data into two groups: (i) a long-term endemic region [9] which includes the Hudson Valley of New York, southern New England, and northern New Jersey (Fig. 1b), and (ii) a more recently endemic region, which includes the rest of the counties in the northeastern region (Fig. 1a). Island counties were excluded from our analyses. Comparisons between these regions allowed us to explore how factors affect inter-annual trends in Lyme disease incidence in regions with presumed stabilized (long-term endemic), and increasing (recently endemic) Lyme incidence. Lyme disease case counts were corrected for county populations using annual county population estimates collected by the United States Census Bureau [45]. All Lyme disease incidence data are presented as the number of cases per county per 100,000 residents.

### Body burden on small mammals

Measurements of the body burdens for *I. scapularis* nymphs and larvae on chipmunks (*Tamias striatus*) and white-footed mice (*Peromyscus leucopus*), respectively, provided metrics for the number of ticks feeding on small mammals. Field data were collected in a long-term study at the Cary Institute of Ecosystem Studies in Millbrook, Dutchess County, NY (41°47′1.04″N, 73°43′56.49″W). The field sites and methods are described in detail in Ostfeld et al. [37]. These data were collected annually from 1994 to 2012 on six small mammal trapping grids. Each trapping grid contained 242 Sherman traps arranged in pairs on an 11 × 11 grid, with 15 m spacing between each trapping station, and each grid covering approximately 2.25 ha. Small mammals were trapped on each grid for two consecutive nights, multiple times between April and November each year. Additional details regarding the small mammal data collection methods are described in Levi et al. [46].

The number of larvae and nymphs were counted on the head and neck of each animal the first time they were caught in a trap. Counting nymphs on the heads of chipmunks provides a reliable metric for their body burdens (number of attached ticks per chipmunk), while counts for the number of larvae on white-footed mice provides a reliable estimate of their total body burden [46]. We calculated the annual average body burden of chipmunks during the *I. scapularis* seasonal nymph peak, and the annual average body burden of white-footed mice during the larval peak. These served as metrics for the number of nymphs and larvae feeding on animal hosts in a given year. The number of larvae counted on the heads of chipmunks, and nymphs counted on the heads of mice did not provide reliable metrics for the animal’s total body burdens, so these data were not used for our analyses [46]. All data collected on the six grids were combined to represent an overall annual average body burden for mice (larvae), and chipmunks (nymphs). All small mammal handling procedures were approved by the Cary Institute of Ecosystem Studies IACUC.

### Density of questing nymphs

The density of questing nymphs was measured at the Cary Institute of Ecosystem Studies during the same time period (1994–2012) and on the same six sites as the small mammal body burden data. Actively questing *I. scapularis* nymphs were collected using a dragging method whereby a 1 m^2^ white corduroy cloth is dragged along the surface of the leaf litter and understory vegetation. All sites were sampled multiple times during the nymphal peak each year (May - July). The total area covered for each sample was 450 m^2^. Drag cloths were checked for ticks every 30 m and the number of *I. scapularis* nymphs was recorded. We averaged the density of *I. scapularis* nymphs during their activity peak across all six sites to estimate an overall density of nymphs (DON) for each year.

### Temperature and precipitation data

County-wide temperature and precipitation data were collected from the CDC’s Wide-ranging Online Data for Epidemiological Research (WONDER) database [47]. The WONDER database provides climate data from National Oceanographic and Atmospheric Administration (NOAA) weather stations in the United States, which can be averaged by various administrative boundaries. We used the mean of all daily temperature and precipitation data collected by weather stations within county boundaries to calculate our parameters. Daily maximum temperatures and daily precipitation were retrieved from the data base for the *I. scapularis* nymph questing period (May - July) for the year the Lyme disease cases were reported. We also retrieved temperature and precipitation data for the previous year’s larval questing period (August - September). We then calculated a cumulative measure for the number of days where T > 25 °C and precipitation = 0 during the previous year’s larval questing period, and the current year’s nymphal questing period. This provided us with metrics for the number of hot, dry days (HDD) during the questing periods of the two immature *I. scapularis* life stages. We abbreviated the metric for the nymphal questing period as N-HDD, and the metric for the larval questing period during the preceding year was L-HDD. Although ideally atmospheric humidity data would be used for this purpose, no consistent daily records exist at the county scale for our study period.

We also compared the field data on tick abundance collected at the Cary Institute of Ecosystem Studies against the CDC WONDER data for Dutchess County between 1994 and 2011. We had one additional year of field data (2012) which was not included in the CDC WONDER database. To calculate N-HDD for 2012 we accessed the NOAA database which is used to calculate the CDC WONDER data, and downloaded weather station data for stations within Dutchess County to calculate maximum temperature and precipitation for 2012 N-HDD.

### Data analyses

We used two mixed effects generalized additive models to explore whether time was a linear estimator for Lyme disease incidence, first in our long-term endemic subset which included the Hudson Valley, Southern New England, and northern New Jersey, and then in the recently endemic subset which included the remaining counties in the northeastern region. Lyme disease incidence is strongly spatially correlated at this scale, so both models included a smoothing term for interactions between latitude and longitude to account for the spatial variation in incidence. The CDC’s reporting metric (which changed in 2008) was also included in both models, as was county as a random effect. County was included as a random effect because observations within the same county are not independent. These factors de-trended the data, and will be referred to hereafter as the de-trending model. We used the de-trending models to explore whether Lyme disease incidence was increasing over time in our two data subsets (long-term endemic and recently endemic) (Fig. 1). We also used these models to explore the effect of summer temperature and precipitation during *I. scapularis* questing periods (L-HDD and N-HDD) on inter-annual variation in our long-term and recently endemic subsets. F-tests and Bayesian information criterion (BIC) values were used to compare models and evaluate the addition of new factors using the procedures described in Zuur et al. [48]. Lyme disease incidence data were log-transformed for all analyses.

We used three linear models to explore the effect of summer temperature and precipitation (L-HDD and N-HDD) on the body burdens of small mammals. The first model included L-HDD compared against the average annual larval body burden of mice in the same year, the second compared L-HDD against the nymphal body burden of chipmunks the following year, and the third compared N-HDD against the nymphal body burden of chipmunks in the same year. All three models included the number of mice or chipmunks caught that year as a covariate. These models allowed us to explore whether summer climate affected the number of ticks feeding on small mammals, and whether that effect carried over from the previous year.

Additionally, two linear models explored the effect of summer temperature and precipitation on the density of questing *I. scapularis* nymphs (DON) using the dragging data collected at the Cary Institute of Ecosystem Studies. The first model compared L-HDD against DON to explore whether the weather during the previous year’s larval questing period affected the number of actively questing nymphs the following year. The second model compared N-HDD against DON to see if summer weather during that year affected nymphal questing activity. These models also included the number of chipmunks caught that year as a factor. Chipmunk count was included as they act as the primary host for nymphs, and chipmunk population density can affect DON [46]. All analyses were performed in R version 3.2.3.

## Results

### Incidence of Lyme disease

**Fig. 2:**
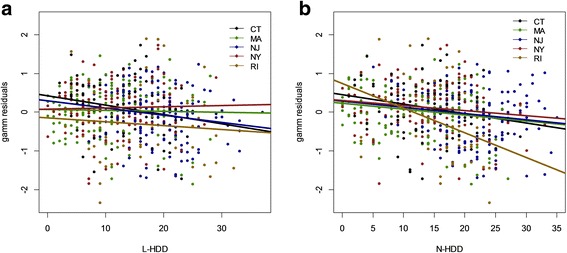
**a** The residuals of the de-trending generalized additive mixed model (gamm) for the long-term endemic region plotted against L-HDD, and **b** the residuals of the de-trending gamm plotted against N-HDD. Best-fit lines are included for each of the five states (Connecticut, Massachusetts, New Jersey, New York, and Rhode Island) included in the model. The de-trending models included CDC reporting type, a smoothing term for latitude and longitude, and county as a random effect

In both the long-term and recently endemic regions inclusion of a smoothing term for latitude and longitude, CDC reporting type, and county as a random effect improved the models based on their BIC scores. Overall, the recently endemic region showed a significant increase in Lyme disease incidence between 2000 and 2011 (*t* = 13.48; *df* = 2210; *P* < 0.001) (Table 1) (Fig. 1a), while time was not a significant linear predictor of Lyme disease incidence in the long-term endemic region (Table 2) (Fig. 1b). We examined the effect of the number of hot (T > 25 °C), dry (Precip = 0) days during the previous year’s larval questing period (L-HDD) and during the current year’s nymphal questing period (N-HDD) on the incidence of Lyme disease in each region in de-trending models (Table 3). L-HDD did not affect the incidence of Lyme disease in either the long-term (*t* = -0.52; *df* = 396; *P* = 0.60) (Fig. 2a), or the recently (*t* = 0.24, *df* = 2209, *P* = 0.82) endemic regions. This conclusion (Fig. 2) is based on the analysis of residuals of the de-trending model for the long-term endemic region plotted against N-HDD and L-HDD. N-HDD had a significant negative effect on the incidence of Lyme disease (*t* = -5.48; *df* = 396; *P* < 0.001) in the long-term endemic region (Fig. 2b). N-HDD had no significant effect on Lyme disease incidence in the recently endemic region (*t* = -0.01, *df* = 2055, *P* = 0.33). The lack of effect of L-HDD on Lyme disease incidence in both regions is also reflected in their BIC scores (Table 3).

**Table 1:** The *F*-test statistics for all the fixed effects included in the generalized additive mixed models for the recently endemic region

Recently Endemic Region			
Parameters	*df*	*F*	*P*-value
Reporting Type	1	43.03	< 0.001^a^
Year	1	175.02	< 0.001^a^
Latitude × Longitude	20	14.16	< 0.001^a^
N-HDD	1	3.67	0.06
L-HDD	1	0.12	0.73

County was included as a random effect in this model. ^a^denotes a factor which significantly improved the model according to the BIC value

Residual degrees of freedom = 2219, *r* ^2^ = 0.52

**Table 2:** The *F*-test statistics for the parameters included in the generalized additive mixed models for the long-term endemic region

Long-Term Endemic Region			
Parameters	*df*	*F*	*P*-value
Reporting Type	1	11.44	< 0.001^a^
Year	1	0.00	0.99
Latitude × Longitude	17	6.21	< 0.001^a^
N-HDD	1	21.38	< 0.001^a^
L-HDD	1	3.37	0.07

County was included as a random effect in this model. ^a^ denotes a factor which significantly improved the model according to the BIC value

Residual degrees of freedom = 565, *r* ^2^ = 0.67

**Table 3:** Bayesian information criterion (BIC) values for the generalized additive mixed models run for the long-term and recently endemic regions exploring the effect of summer temperature and precipitation during the previous year’s larval questing period (L-HDD), and that year’s nymphal questing period (N-HDD)

Model	De-trending Model	Year	L-HDD	N-HDD
Long-term endemic region	1140.1	1144.5	1141.7	**1122.4**
Recently endemic region	5976.0	**5818.1**	5825.7	5822.1

The de-trending models included CDC reporting type, a smoothing term for latitude and longitude, and county as a random effect. BIC values show model improvement for L-HDD, N-HDD, and year when added to the de-trending models. Values in bold indicate a significant improvement

**Fig. 3:**
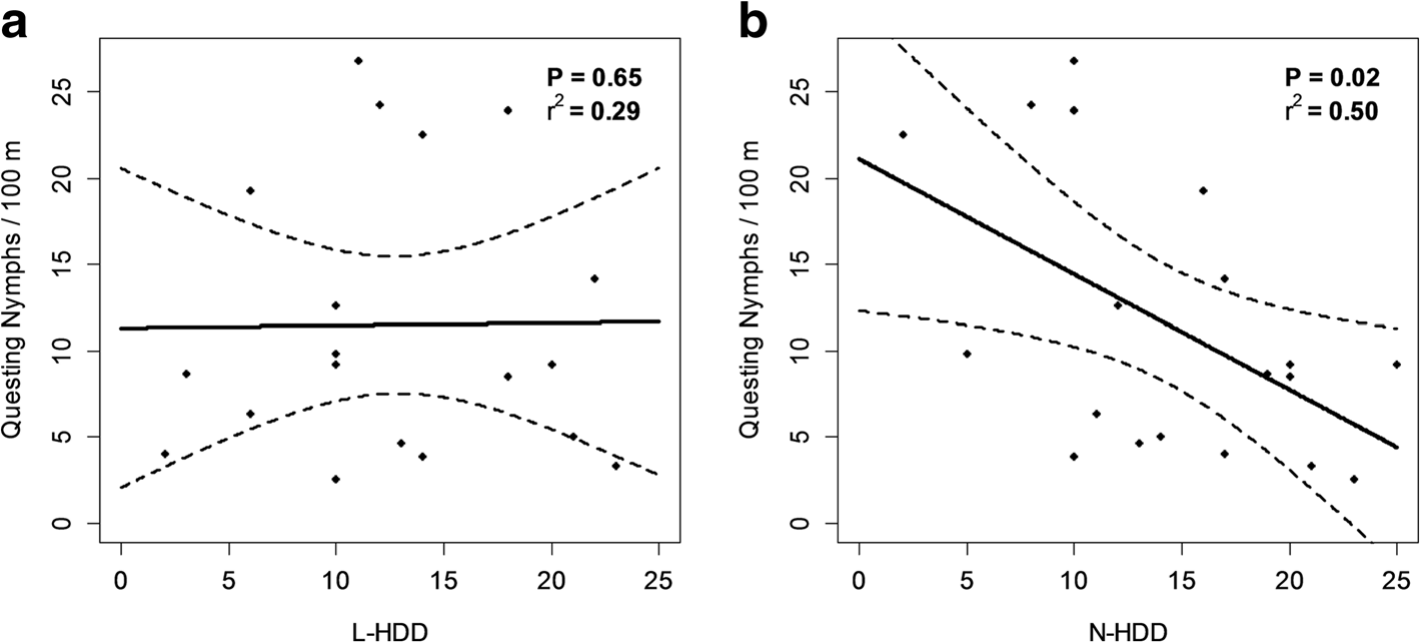
**a** The density of questing nymphs (DON) per 100 m^2^ determined *via* drag sampling compared against L-HDD, and **b** DON compared against N-HDD. Nymphs show lower activity during hot dry summers, but there is no effect of weather from the previous year. *P*-values and *r* ^*2*^ statistics are based on models including both the weather parameters and small mammal counts

L-HDD did not affect the density of questing nymphs (DON) the following year (*t* = -0.46; *df* = 1, 16; *P* = 0.65) (Fig. 3a). On the other hand, N-HDD had a significant negative effect on DON (*t* = -2.60; *df* = 1, 16; *P* = 0.02) (Fig. 3b). The number of chipmunks caught annually over a field season had a significant negative effect on DON in both the L-HDD (*t* = -2.57; *df* = 1, 16; *P* = 0.02), and N-HDD (*t* = -2.69; *df* = 1, 16; *P* = 0.02) models.

**Fig. 4:**
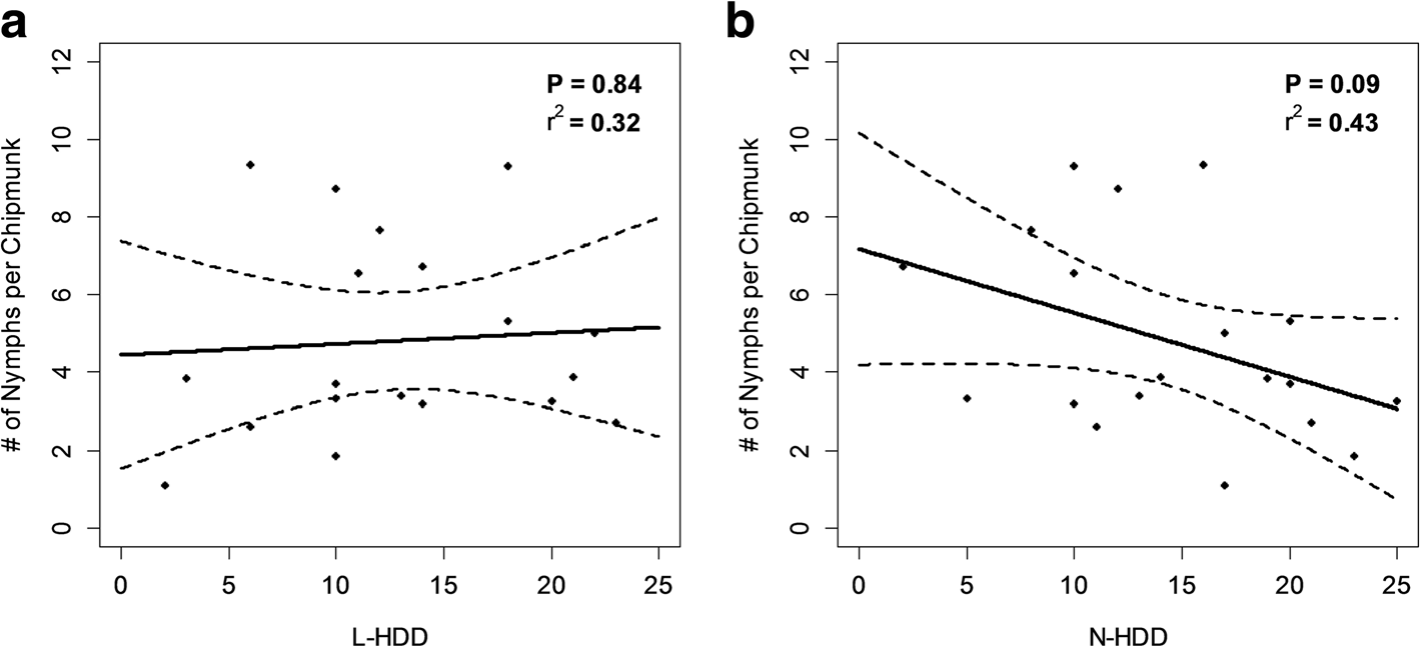
**a** The average body burden of chipmunks during the peak of *I. scapularis* nymph activity plotted against L-HDD, and **b** the average body burden of chipmunks during the *I. scapularis* nymph activity peak plotted against N-HDD. *P*-values and *r* ^*2*^ statistics are based on models including both the weather parameters and small mammal counts. Neither L-HDD nor N-HDD had a significant effect on the number of nymphs found on chipmunks

Summer weather during the questing periods of *I. scapularis* larvae did not affect the average number of larval *I. scapularis* found on mice that year (*t* = -1.56; *df* = 1, 16; *P* = 0.14), or the number of nymphs found on chipmunks the following year (*t* = -0.21; *df* = 1, 16; *P* = 0.83) (Fig. 4a). N-HDD did not have a significant effect on the average *I. scapularis* nymphal body burdens of chipmunks (*t* = -1.82; *df* = 1, 16; *P* = 0.09) (Fig. 4b). The number of chipmunks caught during a given field season had a significant negative effect on the number of nymphs found on chipmunks for both the L-HDD (*t* = -2.70; *df* = 1, 16; *P* = 0.02) and N-HDD (*t* = -2.76; *df* = 1, 16; *P* = 0.01) models. There was a marginally significant negative correlation between the number of mice caught in a field season and the average larval body burden of mice (*t* = -2.09; *df* =1, 16; *P* = 0.05).

## Discussion

### Lyme disease incidence and region

The significant increase in the incidence of Lyme disease in counties in the northeastern United States with a recent endemic history of Lyme disease (Fig. 1a) supports the observation that Lyme disease is spreading rapidly in the United States, with many new areas becoming endemic [9], and showing steadily increasing infection levels [49]. On the other hand, in areas with a long-term endemic history the incidence of Lyme disease appears to have stabilized at least since 2000 (Fig. 1b). Thus, a threshold for Lyme disease incidence appears to exist, above which incidence stabilizes. After this threshold is reached, inter-annual variation in the incidence of Lyme disease may be driven by a different suite of factors that affect the inter-annual variation in tick populations [38], such as weather [24, 50], or host abundance and diversity [37, 51, 52].

In newly emerging areas factors such as physician awareness, human behavior [11, 12], and the amplification of *B. burgdorferi* in host communities [18, 19] may be the dominant factors affecting the recorded human incidence of Lyme disease. When Lyme disease has recently spread to a new area a considerable lag in notifying the public and modifying human behavior may cause an increase in incidence [53, 54]. Physicians may also initially report a high number of cases when Lyme disease is emerging, and become less aggressive in their reporting as it becomes more commonplace [44, 49]. Additionally, Lyme disease is amplified through the natural reservoir and vector communities as new susceptible individuals become infected. During this amplification period it is possible that the inter-annual effects of summer weather on Lyme disease incidence is obscured by the overall increase in pathogen transmission. Detection of weather effects therefore may be more difficult in areas with newly emerging tick-borne diseases.

It is unlikely that all counties in the region will stabilize at the same level of Lyme disease incidence. Factors such as human population density, availability of suitable tick habitat, and human contact rates can affect both *I. scapularis* densities and Lyme disease risk [55–57]. Additionally, physicians often under-diagnose diseases once the disease is fully established in a new region [49] and the under-reporting of Lyme disease cases by physicians in the United States is well-documented [44], which further obfuscates the measurement of this threshold. These factors are all likely to vary among counties, affecting the threshold at which Lyme incidence stabilizes. Further research is needed to explore how these factors might interact to affect the stabilization of Lyme disease incidence in different locations.

### The effect of N-HDD on Lyme disease incidence and DON

We found that weather conditions during the questing period of *I. scapularis* nymphs (N-HDD) affected county-wide incidences of Lyme disease in the long-term endemic region; hot dry summers were associated with significantly reduced incidences of Lyme disease. In contrast, this pattern was not detected in the counties that have more recently become endemic for Lyme disease. This observation supports the assertion that other (non-weather related) factors could mask the effects of weather on inter-annual variation in areas where the disease is newly emerging. Thus, some of the discrepancies that have been observed between studies of inter-annual variation in Lyme incidence in the northeastern United States may be attributed to the length of time since the emergence of Lyme disease in each location [20]. It is notable that the suppressive effect of hot, dry weather was detected despite the fact that human behavior strongly impacts Lyme disease incidence [58], and people spend more time outside during dry weather [59], potentially increasing overall contact rates with ticks. Our detection of a relationship between summer weather conditions and Lyme disease incidence in the long-term endemic region, despite a variety of likely confounding factors, suggests a strong effect of summer weather conditions on *I. scapularis* questing behavior. The effect of weather conditions during the nymphal questing period on Lyme disease incidence may be explained in part by the geotropic response of questing nymphs to drought; when conditions are warm and dry ticks quest at lower heights [42], likely reducing contact rates with humans.

Exploration of trends in the field data for the density of questing *I. scapularis* nymphs collected between 1994 and 2012 supports this hypothesis. We found that there was a strong negative correlation between N-HDD and DON (Fig. 3b), likely due to the effect of vapor pressure deficit on tick activity [34]. Field evidence suggests that when conditions are hot and dry *I. scapularis* alters its behavior, reducing its questing height [22]. Moreover, laboratory evidence also indicates that relative humidity affects *I. scapularis* questing height [42]. This change in behavior is likely to affect contact rates between large animals (including humans) and ticks.

### The effect of L-HDD on Lyme disease incidence and DON

We found no significant relationship between the weather conditions during the previous year’s larval *I. scapularis* questing period (L-HDD) and inter-annual variation in Lyme disease incidence in either region (long-term endemic/recently endemic). Nor did we observe any effect on the density of questing nymphs (DON) the following year. Furthermore, there was no relationship between summer weather conditions and the number of larvae found feeding on mice. This is important as the number of larvae successfully feeding on hosts would have a strong impact on nymph populations the following year [37, 60]. This lack of inter-annual effect was also observed by Berger et al. [36] where weather conditions during previous years did not affect tick densities in the current year. The absence of trends carrying over from the previous summer suggest that the effect of hot, dry summers on tick densities is behavioral, or possibly demographic in the short term, with its effect on Lyme disease incidence restricted to the current season. Overall, we found no evidence of any long-term effect of summer weather on tick populations as a whole, despite a strong short-term effect on the human incidence of Lyme disease and *I. scapularis* nymphal questing activity. Perhaps other factors play a stronger role in the overwinter survival and molting success of larval *I. scapularis*, including variation in host populations [37], or winter precipitation events [24, 25], among others.

### DON and small mammal body burdens

Tick activity or density was significantly reduced during hot dry summers (Fig. 3b), but this trend was far weaker in our analysis of small mammal body burdens (Fig. 4b), probably because of the differential effect of tick behavior on these two metrics of tick abundance. Tick dragging collects actively questing ticks with a relatively high questing height, and is a good measure of entomological risk for humans [61]. This metric is affected by tick questing height because ticks must quest at or above the surface of the leaf litter to come into contact with the collection cloth [50]. On the other hand, the number of ticks counted on small mammal hosts is not as sensitive to changes in tick behavior, and feeding success has a strong effect on tick survival [37, 60]. However, counts of mean tick burdens on hosts can be affected by other factors such as variation in host densities [46], and density-dependent effects whereby tick feeding success is reduced when host body burdens increase [60], although the latter is not consistently observed [62]. The fact that these two methods appear to suggest differing trends may indicate that the impact of hot dry summers on *I. scapularis* is an ephemeral behavioral effect, causing them to quest at lower heights, and may not directly affect tick populations or long-term trends in the incidence of Lyme disease. More research is needed regarding the cumulative effect of long-term drought on *I. scapularis* survival and energetics. Furthermore, the variable results found with differing metrics highlights the importance of using multiple metrics when studying complex vector-borne disease systems.

## Conclusions

Hot, dry weather reduces the density of questing *I. scapularis* nymphs, but does not appear to have a strong impact on the density of *I. scapularis* larvae or nymphs feeding on small mammals. Weather conditions in one summer do not appear to have carryover effects on tick density the following summer. We conclude that summer weather conditions affect *I. scapularis* questing behavior, but may not have strong demographic effects. Reduced density of questing *I. scapularis* nymphs and behavioral effects during hot, dry periods appears to reduce the incidence of Lyme disease in the human population in areas with a long Lyme disease endemic history (long-term endemic region). Similar suppression of nymph density and Lyme disease incidence in hot, dry summers was not detected in areas where Lyme disease is invading (recently endemic region). We suspect that these weather effects are masked in recently endemic areas experiencing directional changes in tick populations, host communities, or human recognition of Lyme disease risk. These regional differences should be taken into account in future modeling attempts.

## Abbreviations

DON: Density of questing nymphs
L-HDD: Number of hot (T > 25 °C), dry (precip = 0) days during the *I. scapularis* larval questing period the previous year
N-HDD: Number of hot (T > 25 °C), dry (precip = 0) days during the *I. scapularis* nymphal questing period
